# Near-infrared spectroscopy and machine learning-based technique to predict quality-related parameters in instant tea

**DOI:** 10.1038/s41598-022-07652-z

**Published:** 2022-03-09

**Authors:** Xiaoli Bai, Lei Zhang, Chaoyan Kang, Bingyan Quan, Yu Zheng, Xianglong Zhang, Jia Song, Ting Xia, Min Wang

**Affiliations:** 1 Present Address: State Key Laboratory of Food Nutrition and Safety, Key Laboratory of Industrial Fermentation Microbiology, College of Biotechnology, Tianjin University of Science and Technology, Tianjin, 300457 China; 2 State Key Laboratory of Core Technology in Innovative Chinese Medicine, Tasly Pharmaceutical Group Co., Ltd., Tianjin, 300410 China; 3 Jiangxi Discipline Inspection and Supervision Technical Support Center, Nanchang, 330036 China

**Keywords:** Computational biology and bioinformatics, Mathematics and computing

## Abstract

The traditional method for analyzing the content of instant tea has disadvantages such as complicated operation and being time-consuming. In this study, a method for the rapid determination of instant tea components by near-infrared (NIR) spectroscopy was established and optimized. The NIR spectra of 118 instant tea samples were used to evaluate the modeling and prediction performance of a combination of binary particle swarm optimization (BPSO) with support vector regression (SVR), BPSO with partial least squares (PLS), and SVR and PLS without BPSO. Under optimal conditions, Rp for moisture, caffeine, tea polyphenols, and tea polysaccharides were 0.9678, 0.9757, 0.7569, and 0.8185, respectively. The values of SEP were less than 0.9302, and absolute values of Bias were less than 0.3667. These findings indicate that machine learning can be used to optimize the detection model of instant tea components based on NIR methods to improve prediction accuracy.

## Introduction

Instant tea utilizes the highest quantity of tea raw materials worldwide, and its consumption has increased rapidly in recent years^1^. The manufacturing process of instant tea primarily comprises extraction, filtration, vacuum concentration, and drying^2^. Instant tea maintains the nutritional characteristics and flavor of traditional tea. In addition, instant tea offers drinking convenience, has low amounts of pesticide residues, and is easy to transport. Consequently, it is popular among consumers and has a broad market prospect^3^. As consumers pay increasing attention to the quality of instant tea, its quality control has also become increasingly important^4^.

The quality of instant tea is determined by several main compounds, specifically moisture, caffeine, tea polyphenols, and tea polysaccharides^5^. These compounds not only give the tea a unique taste, but also provide a variety of health benefits^6^. If the moisture content is too high, the instant tea can produce mildew, and consequently, its nutrition and flavor may change. Therefore, a specific moisture content limit should be maintained during the processing and storage of instant tea to ensure the stability of its quality^7^. Caffeine is an alkaloid with therapeutic effects on many diseases, including metabolic syndrome, type 2 diabetes, liver diseases, and cardiovascular and cerebrovascular diseases^8,9^. Additionally, caffeine contributes a bitter taste to instant tea^10^. Tea polyphenols consist of four major groups: catechins, phenolic acids, flavonoids, and anthocyanins^11^. They have a variety of physiological effects, such as antioxidation, antiradiation, antiaging, hypoglycemic, and bacteriostatic effects^12^. The astringent and bitter taste of tea mainly results from tea polyphenols^13^. Tea polysaccharides, which are acidic, have health benefits, such as lowering blood sugar, blood lipids, and blood pressure; they also enhance the immune system and resistance to hypoxia^14^. Tea polysaccharides can weaken the bitter taste and astringency and alleviate the stimulating effect of tea^15^.

Currently, the conventional physical and chemical methods for determining the levels of moisture, caffeine, tea polyphenols, and tea polysaccharides in instant tea mainly involve oven drying, spectrophotometry, and high-performance liquid chromatography (HPLC)^16–19^. Although techniques based on sizable equipment provide various reliable protocols with good accuracy and sensitivity, they usually suffer from shortcomings such as complicated pretreatment procedures, time-consuming operations, high cost, and a need for professional operators^20^. Therefore, optical spectroscopic techniques are increasingly used for the rapid, nondestructive assessment of food products^21^. Near-infrared (NIR) spectroscopy is particularly attractive for this purpose. The NIR spectral region is mainly the frequency-doubled and combined-frequency absorption regions of the hydrogen-containing group X–H (X being an element such as O, N, S, or C). Because various organic substances contain different groups, and various groups have different absorption wavelengths for NIR light in different chemical environments, the NIR spectrum can be utilized to perform qualitative and quantitative analyses in food component analysis^22^. Since recent years, NIR spectroscopy is being applied in the prediction of tea composition^23^. It has been used to discriminate the roast green tea from different origins, estimate the fermentation degree of Pu'er tea in processing, quantitatively determine the contents of total polyphenols, caffeine, and catechins in tea leaves, and classify special-grade green tea. However, studies performing nondestructive quantitative analysis of biochemical components in instant tea are scant^24^.

In the multivariate data analysis step, the partial least squares (PLS) model is the most widely used model for the quantitative analysis of NIR spectroscopy. Support vector regression (SVR) is also a crucial quantitative analysis algorithm. In recent years, metaheuristic algorithms have been widely adopted as global optimizer methods^25^. The particle swarm optimization (PSO) algorithm is one of these methods; it selects the feature subset, optimizes the model parameters, represents less overhead in operation, and has easier implementation and faster convergence during optimization than other metaheuristic algorithms^26^. Kennedy proposed PSO in 1995 and binary particle swarm optimization (BPSO) of discrete space in 1997^27^. Combined with other classification algorithms, BPSO can obtain improved results.

In this study, we used BPSO respectively with SVR (BPSO–SVR) and PLS (BPSO–PLS) to enhance the randomness of the mutation after the reset mechanism and to keep the particle active in continuous optimization. In addition, a fast experiment for determining moisture, caffeine, tea polyphenols, and tea polysaccharides in instant tea was carried out using different models. This study provides a reference for NIR spectroscopy combined with multivariate statistical analysis to determine food components.

## Methods

### Materials and instruments

A total of 118 varieties of instant tea were provided by Yunnan Tasly Deepure Tea Group Co., Ltd (Yunnan, China). All methods were performed in accordance with the relevant guidelines and regulations. These instant tea is a kind of fine powder solid tea product, which is processed by extracting and drying the tea as raw material. A caffeine standard was purchased from China Institute for Food and Drug Control; acetonitrile and ethanol were purchased from Merck Co., Ltd; phenol and concentrated sulfuric acid were purchased from Chinese Medicines Holdings Co., Ltd; and glucose was purchased from Sigma-Aldrich Chemical Co., Ltd. Unless otherwise specified, all chemicals used were of analytical grade.

A U-3010 UV–Vis Hitachi spectrophotometer (Tokyo) was used to determine absorbance. An Agilent 1260 Infinity HPLC system was used to determine caffeine content. NIR spectrometry was carried out using a Thermo Fisher Antaris II (USA).

### Determination of main components

The moisture content of the instant tea was determined according to ISO 7513:1990. The caffeine content was determined according to ISO 10727:2002 and the tea polyphenol content was determined according to ISO 14502-1:2005. The tea polysaccharide content was determined using a modified phenol–sulfuric acid method^19^.

### Spectral data acquisition

NIR spectra were collected in reflectance mode. Each spectrum consisted of an average of 78 scans, in the range of 10,000–4000 cm^−1^. Before scanning, the instrument was fully preheated for more than half an hour. Three spectra were collected from each sample, and the average spectrum of the three spectra was taken as the original analytical spectrum of that sample. In this study, the spectral pretreatment method used was the standard normal variate transformation (SNV) method. This method removes physical spectral information resulting from particle size.

### Correction set sample division

The acquired spectral data and the reference chemical data were separated into two sets: a calibration set and a prediction set. It has been reported that the tenfold cross-validation method, also called Rotation Estimation, is a practical method to statistically cut the data sample into smaller subsets. The advantage of this method is making full use of small sample data sets^28^. In this study, tenfold cross-validation was used to randomly select the prediction set, and the remaining samples were selected for the calibration set. In each execution, the model was trained using 90% of the data points and tested using the remaining 10%. Therefore, every data point was taken nine times for training and once for testing the model.

### Chemometrics method

#### SVR

The SVR model is mainly used to realize linear regression by mapping spectral data to high-dimensional space and constructing a linear decision function in high-dimensional space^29^. A linear SVR classifier was trained based on the *fitcsvm* function in the Statistics and Machine Learning Toolbox™. Usually, a model selection procedure is required to determine the adjusting parameter *C* to improve the classification accuracy. Because the purpose of this study is to evaluate the search algorithm for spectral data selection, rather than the parameter selection for SVR classifiers, we adopted the default parameter value, i.e., *C* = 1.

#### PLS

PLS is an extensively used class of statistical methods that includes regression, classification, and dimension reduction techniques^30^. It uses latent variables, which are also called score vectors, to model the relationship between input and response variables. In the case of regression problems, PLS first generates the latent variables from the given data and uses them as new predictor variables. There are different types of PLS based on the techniques employed to extract the latent variables.

#### BPSO

The BPSO algorithm transforms the trajectories from a continuous space into discrete space and maintains a swarm of particles and a global best solution simultaneously. In BPSO, each bit only takes a value of “0” or “1,” and the velocities that affect particle positions are transformed into [0, 1] and a stochastic construction process is added to confirm the locations^31^.

### Model evaluation

The performance of the final models was evaluated according to the root mean square error of calibration (RMSEC) and the root mean square error of the verification set (RMSEP). The optimal model method was chosen based on RMSEC and RMSEP as the index which were lower and close to each other. At the same time, the correlation coefficient of validation set (Rp), bias-corrected standard error of prediction (SEP) and Bias were used as auxiliary reference indexes for model evaluation^32,33^.

### Software

Data processing and modeling analysis was carried out using MATLAB 2014a.

## Results and discussion

### Spectra investigation

**Figure 1:**
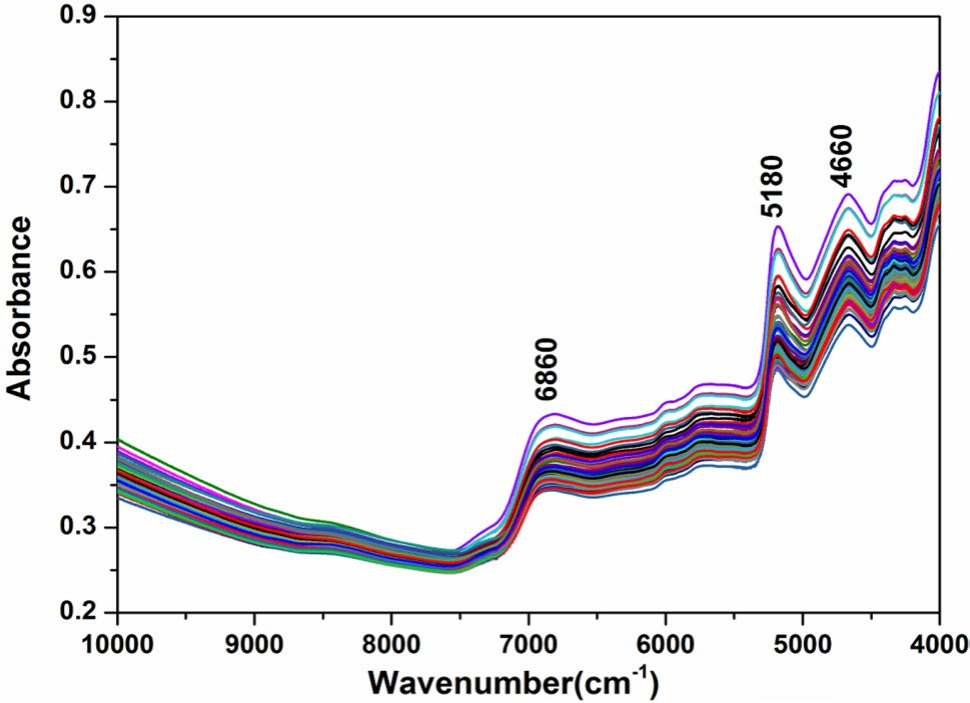
Original near-infrared spectra of 118 instant tea samples.

The original NIR spectra of the 118 instant tea samples are shown in Fig. 1. These spectra can reflect the intrinsic quality of the instant tea samples. The instant tea samples were similar in their type and place of production, and their NIR spectra were understandably similar as well. The first frequency-doubling peak of the N–H bond stretching vibration was at 6860 cm^−1^. The second frequency-doubling peak of the C=O stretching vibration was at 5180 cm^−1^, and the combined-frequency peak of the primary amine and tertiary amine stretching vibration was at 4660 cm^−1^ (Fig. 1). The spectral characteristics depend on the sample composition and provide a theoretical basis for the rapid prediction of moisture, caffeine, tea polyphenols, and tea polysaccharide contents.

### Classification of sample sets and distribution of measured values

A quantitative analysis of moisture, caffeine, tea polyphenols, and tea polysaccharides was carried out on the 118 samples. The results in Table 1 show that the ranges of moisture, caffeine, tea polyphenols, and tea polysaccharide content in the samples are 3.76–6.29%, 0.48–2.80%, 18.70–22.40%, and 18.00–24.50%, respectively. The tenfold cross-validation method was used to randomly select the prediction set, and the remaining samples were selected for the calibration set.

**Table 1:** Sample composition content statistics.

Index	Min%	Max%	Mean%	Standard deviation%
Moisture	3.76	6.29	4.79	0.60
Caffeine	0.48	2.80	1.69	1.65
Tea polyphenols	18.70	22.40	20.93	0.89
Tea polysaccharides	18.00	24.50	21.18	2.17

### Modeling Results

It has been reported that BPSO algorithm based on the traditional machine learning algorithm have a positive impact on the results of the model prediction^34^.The BPSO method was used to optimize the parameter combination, obtain the best tenfold cross-validation accuracy, and establish the model with the strongest prediction ability. In the BPSO process, the relevant parameters were set as follows: the swarm size was 20, the learning factors C_1_ and C_2_ were 2, the maximum evolutionary algebra was 100, and the weight parameter |V_max_| = 6. The results in Table 2 show that the SVR, BPSO–SVR, PLS, and BPSO–PLS models could predict the moisture, caffeine, tea polyphenols, and tea polysaccharides of instant tea. The results showed that RMSEC and RMSEP presented a lower value by BPSO algorithm than those by SVR and PLS alone, which indicate that the addition of BPSO algorithm can improve the accuracy of model prediction. In addition, based on RMSEC and RMSEP, most of algorithm values between the calibration set and the prediction set in BPSO-PLS model were lower than those in BPSO-SVR model, and the range of SEP and Bias values were reasonable, which showed that BPSO-PLS model was stable.

**Table 2:** Comparison of quantitative models for moisture, caffeine, tea polyphenols, and tea polysaccharides in instant tea.

Component	Modeling method	Rc	RMSEC	R_P_	RMSEP	SEP	Bias
Moisture	SVR	0.9852	1.3512	0.9028	1.0117	0.3297	− 0.4412
	BPSO–SVR	0.9884	1.189	0.9710	0.6670	0.3350	− 0.1934
	PLS	0.9552	2.0706	0.9419	0.8123	0.1880	− 0.1264
	BPSO–PLS	0.9983	0.4128	0.9678	0.6293	0.2230	− 0.2272
Caffeine	SVR	0.9909	1.105	0.8514	1.2096	0.3076	0.0619
	BPSO–SVR	0.9916	1.0792	0.9610	0.6728	0.1548	0.0056
	PLS	0.9661	1.714	0.9596	0.6205	0.2484	0.1017
	BPSO–PLS	0.9981	0.4145	0.9757	0.5114	0.2647	0.1027
Tea polyphenols	SVR	0.9579	2.8418	0.6482	2.3088	1.0408	− 0.9307
	BPSO–SVR	0.9594	2.8273	0.7948	2.0272	0.7084	− 0.5186
	PLS	0.7391	6.1777	0.7022	2.1779	0.6531	− 0.4879
	BPSO–PLS	0.9960	0.8191	0.7569	2.1082	0.7233	− 0.3667
Tea polysaccharides	SVR	0.9438	7.2186	0.6621	4.8339	0.8461	0.1784
	BPSO–SVR	0.9465	7.1464	0.8040	4.1831	0.8090	− 0.0615
	PLS	0.7804	13.0553	0.7558	4.5883	0.8207	− 0.3354
	BPSO–PLS	0.9954	2.0187	0.8185	4.3109	0.9302	− 0.0980

**Figure 2:**
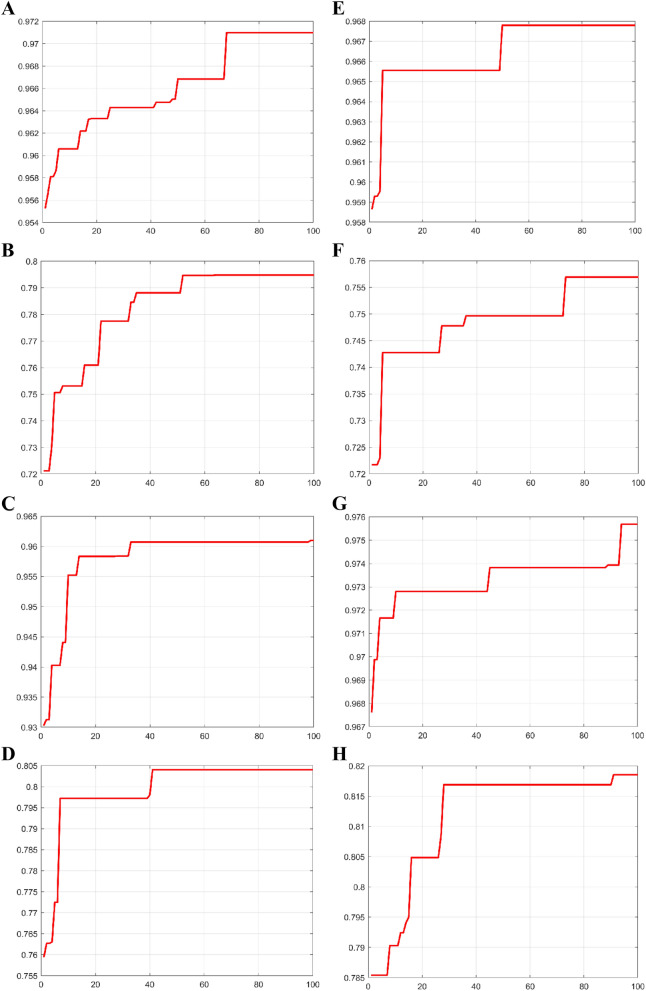
(**A**–**D**) Parameter optimization results of the SVR model based on BPSO with fitness value versus number of iterations: (**A**) moisture, (**B**) caffeine, (**C**) tea polyphenols, and (**D**) tea polysaccharides. (**E**–**H**) Parameter optimization results of the PLS model based on BPSO with fitness value versus number of iterations: (**E**) moisture, (**F**) caffeine, (**G**) tea polyphenols, and (**H**) tea polysaccharides.

Figure 2 shows the convergence curve of the BPSO algorithm with the best results during the 100 runs. The model shows a large fluctuation at the beginning of the iteration, after which it decays with a small trend.

The BPSO–PLS model showed the most stable comprehensive performance and the most accurate prediction results for moisture. The values obtained for R_c_, RMSEC, R_p_, and RMSEP were 0.9983, 0.4128, 0.9678, and 0.6293, respectively. Comparing the four models and convergence curves for caffeine, the BPSO–PLS model had the most stable comprehensive performance and the most accurate prediction results; the R_c_, RMSEC, R_p_, and RMSEP were 0.9981, 0.4145, 0.9757, and 0.5114, respectively. For tea polyphenols, using the BPSO feature selection algorithm, R showed a significant improvement.The R_c_, RMSEC, R_p_, and RMSEP were 0.9960, 0.8191, 0.7569, and 2.1082, respectively. For tea polysaccharides models, using the BPSO feature selection algorithm, R also showed a significant improvement. The BPSO–PLS model had the most stable comprehensive performance and the most accurate prediction results for tea polysaccharides, and the R_c_, RMSEC, R_p_, and RMSEP were 0.9954, 2.0187, 0.8185, and 4.3109, respectively.

**Figure 3:**
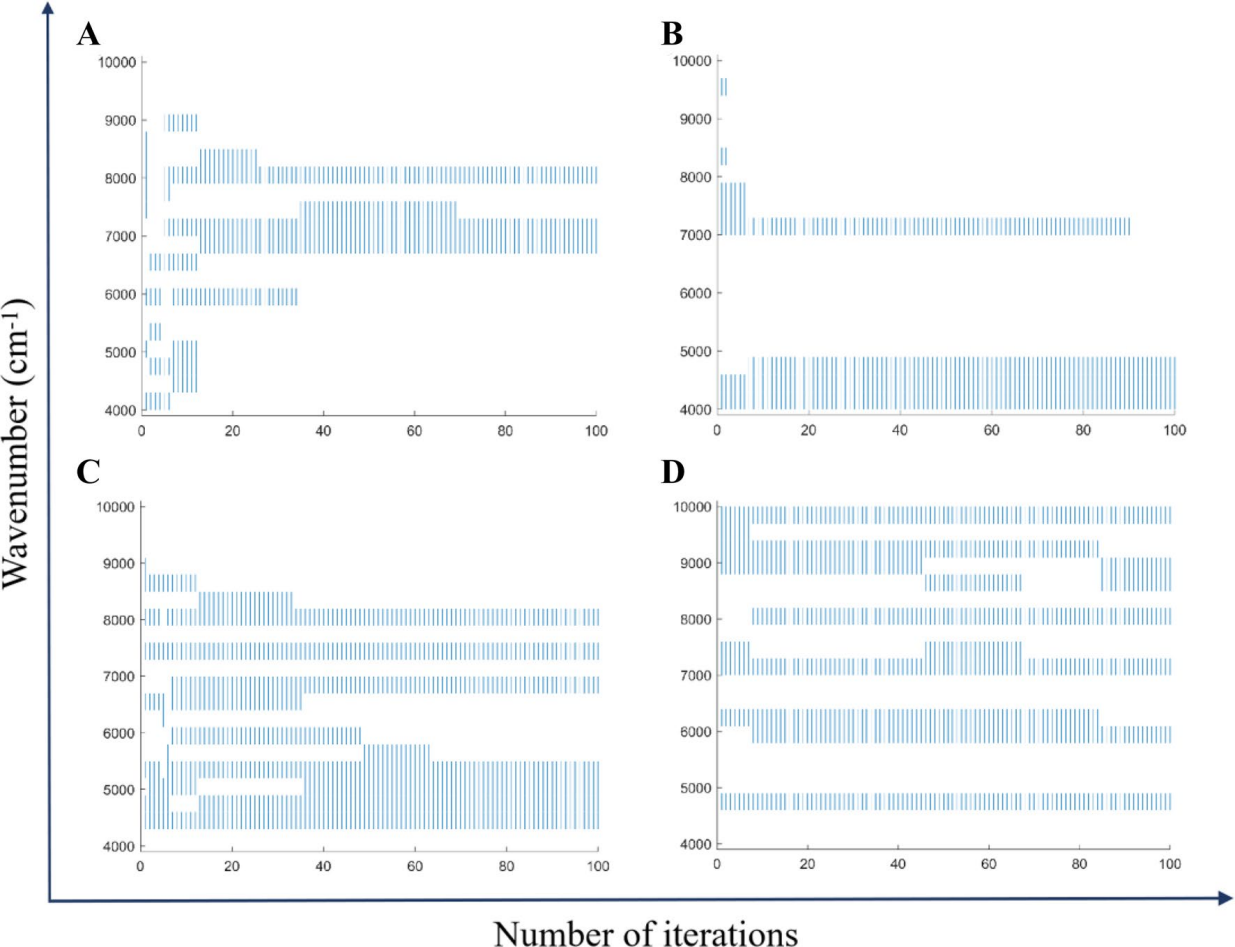
Wavenumber selection results of the four components: (**A**) moisture, (**B**) caffeine, (**C**) tea polyphenols, (**D**) tea polysaccharides.

A spectral range was set, such that if it was selected more than 50 times, then this range was the final selected result. The process of selecting the wavenumber of the four components, resulting from 100 iterations, is shown in Fig. 3. We divided the spectral range into 20 segments with the same width of 311, and the last segment with a width of 319. From the wavenumber, we found that the spectral ranges selected for moisture and caffeine were relatively concentrated, while those for tea polyphenols and tea polysaccharides were relatively scattered.

Table 3 shows the results of selecting the wavenumber of the four components: moisture, caffeine, tea polyphenols, and tea polysaccharides. The characteristic bands of water in instant tea were mainly concentrated in the two wavebands of 6694–7293 and 7892–8193 cm^−1^. The first-order frequency doubling of O–H stretching vibration in pure water is about 7143 cm^−1^, and the combined frequency absorption was 8197 cm^−1,^^35^. The characteristic band of the moisture in instant tea associated with compounds containing O–H group through hydrogen bond in various forms, and further make a shift of the absorption peak in the direction of long and short wavelengths. The characteristic bands of caffeine were concentrated in 4000–4894 and 6994–7293 cm^−1^. Near 4610 cm^−1^ was the combined frequency peak of stretching vibration from primary amine and tertiary amine^36^. In addition, the characteristic bands of instant tea polyphenols were concentrated in 4295–5494, 6694–6994, 7293–7593, and 7893–8193 cm^−1^. 4662 cm^−1^ was the second-order frequency doubling caused by C–C stretching vibration. Near 5000 cm^−1^ was the combined frequency of free O–H stretching vibration in phenols^37^. 6782–6894 cm^−1^ was the first-order frequency doubling of O–H. The characteristic bands of tea polysaccharides in instant tea were mainly concentrated in 4595–4894, 5794–6394, 7001–7293, 7893–8212, 8793–9393, 9692–10,000 cm^−1^. Near 4631 cm^−1^ was the combined frequency absorption peak of the primary amine group, and 4779 cm^-1^ indicated the presence of acyl group. 5333–6154 cm^−1^ was the third-order frequency doubling generated by C–C stretching vibration. The 5714–6667 cm^−1^ range was the vibration region of the amide and carbonyl groups. 6667–8333 cm^−1^ range was the absorption region of protein. The mixed vibration absorption region of fatty acids and polysaccharides was in the range of 8333–10,000 cm^−1,^^38^.

**Table 3:** Results of selected NIR wavenumber of the four components: moisture, caffeine, tea polyphenols, and tea polysaccharides.

Component	Wavenumber (cm^−1^)
Moisture	6694–7293, 7892–8193
Caffeine	4000–4894, 6994–7293
Tea polyphenols	4295–5494, 6694–6994, 7293–7593, 7893–8193
Tea polysaccharides	4595–4894, 5794–6394, 7001–7293, 7893–8212, 8793–9393, 9692–10,000

**Figure 4:**
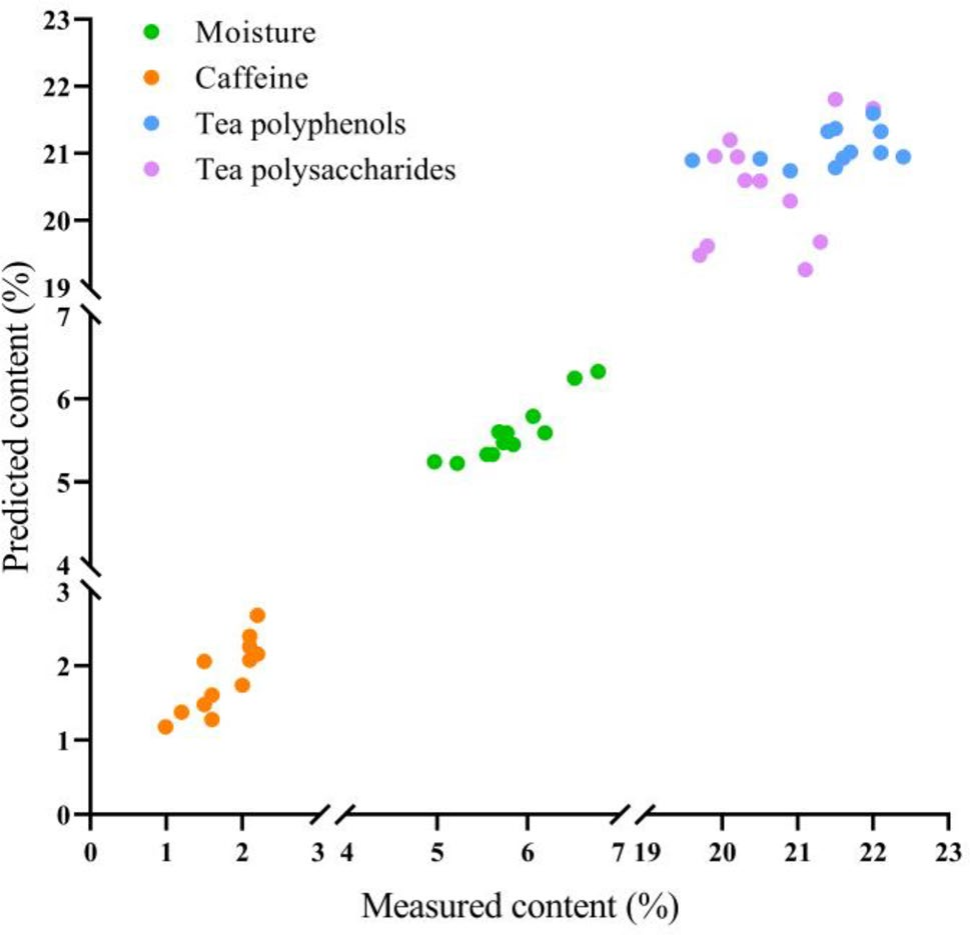
Scatter plots of four components in instant tea samples between actual and predicted NIR values. Orange dots present caffeine, green dots present moisture, blue dots present tea polyphenols, and purple dots present tea polysaccharides.

The scatter plots of 4 components between actual and NIR predicted values were shown in Fig. 4. It is well known that scatter plots present the relationship between two variables in two-dimensional coordinates, which can be used to evaluate the predictive ability of the model. In this study, the scatter points of moisture and caffeine between actual and predicted NIR values were concentrated and close to the diagonal. few scatter points of tea polyphenols and tea polysaccharides are relatively departed from the diagonal due to the complex structure. Taken together, the results indicate that the model exhibits a high prediction accuracy.

## Conclusions

In this study, a rapid NIR method to estimate the moisture, caffeine, tea polyphenols, and tea polysaccharide contents of instant tea was developed using different model calibrations. The tenfold cross-validation method was used to randomly select the prediction set, and BPSO was employed as the optimization algorithm for SVR and PLS. The results show that the R_p_ is above 0.9 for moisture and caffeine, and the R_p_ is approximately 0.8 for tea polyphenols and tea polysaccharides. Therefore, these models exhibited high precision and accuracy. This approach provides, for the first time, a fast, specific, and easily automatable method for the quantitative detection of moisture, caffeine, tea polyphenols, and tea polysaccharides in instant tea samples. This will enable the development of online compositional analysis techniques for more effective process management and quality control.
